# Agency as an Inherent Property of Living Organisms

**DOI:** 10.1007/s13752-024-00471-7

**Published:** 2024-08-14

**Authors:** Bernd Rosslenbroich, Susanna Kümmell, Benjamin Bembé

**Affiliations:** 1 Institute of Evolutionary Biology and Morphology Centre for Biomedical Education and Research, Faculty of Health, School of Medicine, Witten/Herdecke University, Witten, Germany; 2 Alanus University of Arts and Social Sciences, Alfter, Germany

**Keywords:** Agency, Autonomy, Evolution, Organicism, Organismic biology

## Abstract

A central characteristic of living organisms is their agency, that is, their intrinsic activity, both in terms of their basic life processes and their behavior in the environment. This aspect is currently a subject of debate and this article provides an overview of some of the relevant publications on this topic. We develop the argument that agency is immanent in living organisms. There is no life without agency. Even the basic life processes are an intrinsic activity, which we call the organismic level of agency. In addition to this we describe several further levels. These capture different qualities that occur or transform during evolution. In addition to the organismic level, we propose an ontogenetic level, a level of directed agency, directed agency with extended flexibility, and a level that includes the capacities to follow preconceived goals. A further property of organisms is their autonomy. It has been shown that the capacity for autonomy changed during evolution. Here we propose that the two organismic properties autonomy and agency are closely related. Enhanced physiological and behavioral autonomy extends the scope of self-generated, flexible actions and reactions. The increase in autonomy through the evolution of a widened scope of behavioral possibilities and versatility in organisms coincides with extended levels of agency. Especially the human organization, including the sophisticated brain, is the basis for an extended level of agency referring to the capacities to follow preconceived goals. However, it is important for the understanding of the phenomenon of agency not only to assume this latter form, but also to look at the different levels of agency.

## Introduction

We are sitting in a meadow near a babbling brook. It is one of those meadows of our childhood, which were still colorful and diverse in composition, and in which an incomprehensible abundance of beetles, bees, flies, and butterflies cavorted. High in the air a lark is singing in flight, spreading its song across the meadow.

There is activity all around us. The butterflies and the beetles are just the most obvious by their movements. So is the lark, beating its wings at a high frequency, staying in one spot in the air, to perform its song through its stream of breath. However, activity is also on the ground, where insects and snails roam, and over which now and then a mouse scurries through the grass. And it is also activity through which the grass and all the different herbs have been growing. When the meadow really starts to grow in April and May, it goes breathtakingly fast until all the plants stand tall, and the flowers of the many herbs open their blossoms.

Activity is also in the soil. Millions of organisms work the soil mechanically and chemically, forming a complex web of life that only through its activity gives rise to its humus cover. The trout in the brook swims seemingly effortlessly against the current of the water, while the water just flows down the slope, simply along the path of least resistance. The butterflies above the meadow are active on their own and amazingly are able to fly against many a strong breeze, while the clouds above the meadow are pushed forward by the wind.

The immediate impression is that wherever there are living beings, there is an autonomous activity that defies the surrounding physical conditions.

Our main thesis in the following text will be that self-activity, i.e., agency, is immanent in living organisms. There is no life without agency. All basic functions of cells such as metabolism, seclusion from the environment, intracellular transport functions, protein synthesis, organization and usage of information, exchange with the environment, as well as perception of the environment and reactions to it are all activities. These activities need energy and molecular turnovers, as well as information, but they are not reducible to them. With the appearance of multicellular systems new levels of autonomous activities evolved. By building these more complex forms of self-activity on the basis of the basal form of agency of the organism’s cells, hierarchically nested systems emerged. This thesis will be examined more closely in the following text. The basic proposition will be that life does not occur without agency in the first place, and ever more autonomous levels of agency evolved on different evolutionary pathways.

## The Organismic Approach

We base this hypothesis on a consequent organismic approach to living beings (Rosslenbroich 2023a). This approach attempts to take the primary properties of living beings seriously and to propose these properties as the actual starting point for scientific investigations, rather than reducing life functions to mechanisms according to linear cause–effect chains. Living is described as an ontologically distinct reality.

One of these primary properties is that functions and processes in organisms are organized in circular causalities, interdependencies, and networks, generating relationships of interaction. Another property is that organisms establish multilevel, integrative systems, which regulate and organize the functions and the handling of molecules, energy, and information, which in turn partake in the organization of the systems. There is a concurrency between top-down and bottom-up functions in different system levels such as cells, tissues, organs, and the organism as a whole. A further property is the actively organized time structure in organisms. Every organism establishes its specific organization of time and its integration into the time structure of its environment. In the same way we see agency as a primary property of living beings. Together, these properties, which are repeatedly reestablished within ongoing developmental cycles, generate a relative autonomy of every individual organism, because it is more or less extensively regulated and stabilized against the environment.

This organismic approach is based on the results of modern biology, so that there is no need to fall back into vitalistic notions. However, the approach also challenges the widespread claim that organisms can be described by any machine analogy. This applies both to the older analogies conceived along the lines of classical mechanics, as well as to the analogies with computers and computer programs that are so widely proposed today. In this sense, since the beginning of the 20th century organismically oriented authors have perceived their work as exploring ideas between mechanistic and vitalistic views in order to get closer to what organisms really do (Woese 2004; Gilbert and Sarkar 2000; Capra and Luisi 2014; Nicholson 2014; Peterson 2016).

## History

The principle of organismic agency has been excluded from biology for a long time. In her book *The Restless Clock*, Riskin (2016) examines the origins and history of banning agency from science as a property of natural entities. The primary and obvious experience that living organisms are self-active was lost in the context of the increasing imposition of mechanistic thinking since the 17th century.

Riskin unravels a long-standing conflict between those who wanted to outsource agency to a divine engineer and those who reduced agency to the mechanics of component parts of the perceivable organism. In both cases the organism itself is not the active entity but is driven by principles in the background. In this sense, organisms are seen as clockworks, set in motion either by divine intervention or by mechanical forces.

Riskin then shows how these attitudes have been literally woven into the fabric of animal and human science to this day. Against this background, the study and understanding of organismic agency had taken a back seat, although it has been treated, for example, in autopoiesis theories (Roth and Schwegler 1981; Di Paolo 2005; Capra and Luisi 2014), in biosemiotic theories (Hoffmeyer 2008, 2013; Sharov and Tønnessen 2021), or in connection with autonomy theories (Barandarian et al. 2009; Arnellos et al. 2010; Arnellos and Moreno 2015; Moreno and Mossio 2015; Moreno 2018; Virenque and Mossio 2024). Recently, however, there has been an increased interest in this topic resulting in a currently growing number of publications (e.g.Walsh 2015; Aaby and Desmond 2021; Delafield-Butt 2021; Okasha 2021, 2024; Potter and Mitchell 2022; Sultan et al. 2022; Ball 2023a; Corning et al. 2023; Noble and Noble 2023; Moss 2024; Pickering 2024; Watson 2024).

## Agency as a Primary Property of Organisms

Walsh (2015) provides a thoughtful book on the question of agency. He distinguishes between object theories and agent theories. Objects underlie forces, laws, and initial conditions, according to the Newtonian paradigm. Crucially, in the Newtonian paradigm the forces, laws, and initial conditions are extraneous to the objects and exist independently of them. Agent theories, however, are characterized by “immanence” and “explanatory reciprocity.” In an agent theory the entities in the domain include both agents and the principles that are used to explain their dynamics. The agent’s activities are generated endogenously; agents cause their own changes in state in response to the conditions they encounter. These conditions, in turn, are largely of the agent’s making. So, as agents implement their responses to their conditions, they not only alter their own state, they also change the conditions to which their activities are a response. In this sense we present an agent theory.

We define *agency* as the overall *autonomous activity* of the organism to maintain life functions, to establish and defend its processual relative autonomy, and to operate within the environment. It consists in the capacity of the system to perform the processes of its immediate existence as a living organism within a certain self-organized and inherited time structure and to respond actively to internal and external conditions and signals.

This definition includes several aspects. A first aspect is that we include internal life-sustaining activities on the one hand, and engagements with the environment on the other hand within one principle. We hold that both are physiologically interrelated, influencing and constraining one another, so that they basically exhibit the same principle. Below we will propose a differentiation into levels, but these are merely different aspects of one and the same principle.

A second aspect is that agency and autonomy are interrelated, but not the same. In our definition agency focuses on the self-activity that generates the processes and the activity in the environment, while autonomy focuses on the capacity of resilience and flexibility of the organism. However, they are strongly interrelated, as an organism needs agency in order to generate autonomy and establishes autonomy in order to be an agent. So, they are like the familiar two sides of the same coin.

A third point is that we set this organismic principle apart from a causal explanation. Agency as the overall autonomous activity is produced continuously and of its own accord by the organism. Frankfurt (1978) examines this point in his essay beginning with the sentence: “The problem of action is to explicate the contrast between what an agent does and what merely happens to him” (p. 157). However, in our article we will not further discuss the problem of causality, although it is in the background of the epistemological problem of understanding agency. Thoughtful considerations on this point have been provided before (see, for example, Frankfurt 1978; Juarrero 2002; Potter and Mitchell 2022).

Agentic activity, for example, is seen in every living cell, which performs a metabolic turnover. This is not just a series of chemical reactions along an energy gradient, but rather it is performed actively and permanently. The cell arranges all conditions and components in such a manner that the permanent metabolism is possible and in a sophisticated manner integrated into the cell.

Additionally, in multicellular animals circulatory systems are kept running—besides many other functions—and in many there is a heart that beats lifelong. The earthworm digs actively through the soil, fundamentally changing it, birds move through the air, and many animals appear to choose their actions from a repertoire of behaviors. That is, in addition to internal life-sustaining activities, organisms have active engagements with their environments.

Also, the growth of a plant is a general form of agency. In a regulated manner some of its cells perform mitosis and thus add new tissue to the plant again and again. Mitosis is an active process, highly regulated within a sequence of events of several hours within the dividing cells. Plants actively grow contrarily to gravity and roots actively penetrate the soil, even able to activate nutrients from it.

For the moment these statements seem to be simple and self-evident, but they have far-reaching consequences. The conventional view of the organism localizes the ability that something can be active into the physicochemical processes. According to this view, events within a cell or an organism are driven by molecular energetic reactions, as the motor of all functions. Thus, the activity is, more or less explicitly, seen within the metabolic “machinery.”

However, a consequent phenomenological view shows that agency can only come from a whole and intact organism. Minimally, agency needs a functioning cell and its integrity. Only an intact organism can arrange all conditions and components in such a manner that a metabolism and the regulated usage of energy within a surrounded unit becomes possible. An organism keeps its metabolism going, obtains the necessary energy, encloses itself from the environment, and constructs substances such as proteins and nucleic acids.

Organisms establish an energetic gradient toward their environment. The maintenance of this gradient and its defense against the influences from the environment need to be actively performed. At the least it needs a functioning cell, which is not reducible to subcellular functions, structures, or substances, although it uses and needs the metabolic overturn to fuel these activities.

Organisms have often been described from the perspective of thermodynamic disequilibrium (Prigogine and Stengers 1984; Kauffman and Clayton 2006; Penzlin 2014). Thermodynamic equilibria occur spontaneously. In contrast, every living organism permanently prevents itself from relapsing into such a state of equilibrium. For this the organism does not just prevent degradation, but rather permanently balances synthesizing and degrading processes. This balance is not a fixed state, which exists once it is established. Rather it needs to be generated constantly. Every organism exists in such a permanent process of degradation and synthesis, self-generation and self-renewal (Penzlin 2014).

## Activity all Over

Thus, cells as well as multicellular organisms must have continuous activity to ensure their existence and their self-renewal. Since most of these functions repeat continuously and are subject to characteristic variation in their course, they generate periodicities, which are described by chronobiology (Hildebrandt et al. 1987; Dunlap et al. 2004; Koukkari and Sothern 2006; Moser et al. 2008). From this research it is well known today that these periodicities and their time structure are endogenously generated.

Also, the transformation of energy in the cell is subject to its activity and is highly regulated by respective enzymes. Energy from nutrients is not released within one big event but rather in very small quantities, during which the energy is transferred to ATP (Adenosinetriphosphate, derived from Adenosindiphosphate ADP). This guarantees that the energy can be used in many functions and is not just exploding, leaving nothing but heat.

The cells of a multicellular organism are supplied with nutrients, but the transformation of energy into utilizable ATP is performed by the cell itself. Within the inner membrane of the mitochondria, there is an ADP/ATP-translocator which provides the cytoplasm with ATP, and rapidly takes back ADP to the mitochondrial matrix, so that it can again be used to generate ATP. This translocator works very fast. While only small amounts of ATP can be stored within the cell, it has a large turnover rate. Within the human body, each molecule of ADP/ATP switches several thousand times per day between the mitochondrion and the cytoplasm. It has been estimated that all the cells of a resting human together have a turnover rate of ATP of about 1.7 kg per hour. During intensive exercise this amount can increase to 30 kg per hour. This again has a clear character of processual activity.

All organisms, from the most rudimentary to the most complex, exhibit an unresolvable functional relationship between this intrinsic activity and molecular, energetic, and informational processes. Thus, an essential property of a *living organism is an organized*,* active periodic process or a continuous activity*,* and what is being processed are substances*,* energy and information.*

This organismic level of agency (for explanation of levels see below) cannot be reduced to either molecular reactions, energy, or information alone. The organized functioning of these three qualities is a prerequisite of a living cell. The living system is generated by the interaction of its components, and the components are integrated and regulated by the system. This is again one of those interdependencies that are so typical for any living organism. This problem has been discussed in detail in the literature from various points of view and under different concepts. Some previous publications include Gilbert and Sarkar 2000; Woese 2004; Noble 2006, 2017; Henning and Scarfe 2013; Hofmeyr 2017; Ball 2023b.

## Two More Examples

Even the integrity of the plasma membrane is a result of the activity of the cell. The membrane is constantly maintained in a special intermediate status of fluidity. If membranes tend to harden, the cell integrates more phospholipids with unsaturated fatty acids, and when they tend to dissipate it integrates more phospholipids with saturated fatty acids. The cell is also able to regulate membrane fluidity by regulating the amount of cholesterol being integrated. Thus, the cell uses the chemical properties of the bipolar phospholipid molecules. Membranous functions depend on these properties, but to synthesize them and to organize them in a functional membrane underlies the constant activity of the cell (Agutter et al. 2000).

A second example comes from recent insights in genetics. During most of the 20th century the genome was regarded as a program to construct an organism, and as a kind of control unit within the cell. The organism was viewed as a passive vehicle for retaining genes in a “gene pool,” and that the behavior and function of organisms were controlled to this end (Noble and Noble 2023). New insights in genetics, however, show that it is essentially the cell that organizes and uses the genome. It organizes its replication, including refined systems to correct errors. The cell regulates the transcription and all the complex following processes such as splicing, and in many cases it is even able to reorganize and to change the genome according to its needs. In recent years Shapiro (2011, 2013, 2014, 2017) has been pointing vehemently to this aspect. He argues that organisms deal much more actively with their genome than has been previously thought. Organisms also play a much more active role in genomic changes during evolution, adding an impact of biological agency on evolutionary innovation. Shapiro sees the genome more as a “read-write genome” rather than a “read-only genome.”

In addition to these changes, inheritance also occurs on several levels of the organism, not only on the DNA level. Additional levels are the epigenetic, the behavioral, and the symbolic levels (Jablonka and Lamb 2005) which all interact with one another. Noble and Noble (2023, p. 9) conclude:

Inheritance is a process, not a discrete, measurable entity. What we inherit is a propensity to do things. So we inherit not in two discrete parts, cause (gene) and effect (trait). We inherit a capacity of becoming or being, where cause and effect are one.

Physiology in general is a science dealing with activities. Turner (2007, 2013, 2017) postulates that homeostasis is the crucial principle to develop “a coherent theory of life.” Homeostasis, he holds, is generated by self-sustained, active processes, and the capacity to act is where the distinction can be drawn between a living being and nonliving objects of certain resemblances with living beings.

## Autonomy

As depicted above, agency is strongly related to the principle of organismal autonomy. *Organismic agency generates autonomy and autonomy affords agency.*

In the words of Walsh (2015, p. 217):

In constituting their affordances through self-maintaining, self-regulating activities, agents forge for themselves a degree of freedom from the vicissitudes of their environments. In doing so they determine which of the conditions is salient, and what they afford. And they set themselves up to exploit the opportunities those conditions have to offer. This is the sense in which agents are ‘autonomous’.

Living systems establish a relative autonomy in the sense that they maintain themselves in form and function within time and achieve a self-determined flexibility. These living systems generate, maintain, and regulate an internal network of interdependent, energy-consuming processes, which in turn generate and maintain the system. The prerequisites, such as genetic and partly the epigenetic information, but also some cell components, are inherited. Thus, every organism stands in a long series of perpetual cycles of construction and degradation, of reproduction, development, and death. But the rebuilding within the developmental cycle is done actively in each case.

Moreno (2018, p. 292) summarizes very briefly: “a system is autonomous if it actively maintains its identity.” Then he describes a very helpful distinction between constitutive processes, which generate identity and largely delimit what the system actually is, and interactive processes, with the specific function of controlling interactions with the environment (first introduced in Arnellos and Moreno 2015):

As a subset of constitutive processes, interactive processes are generated by and depend on the existence and stability of the whole autonomous organization. In turn, they contribute to the maintenance of that very organization by specifically managing its relations with its environment. (Moreno 2018, p. 292)

However, differently from Moreno and colleagues, for whom constitutive processes (excluding the interactive processes) are not agentic, we see agency already realized in the inner processes of the cell. A single-celled organism establishes a boundary and actively regulates its interaction and exchange with the environment. It specifies its own rules of behavior and reacts to external stimuli in a self-determined way. The cellular membrane encloses a defined reaction room, thus contributing to the maintenance of the cell’s identity and therefore to its autonomy. *Metabolic processes* within the cell construct the boundaries, but the metabolic processes themselves are made possible by those boundaries.

Autonomy is achieved by using *energy-rich molecules*. In the face of the hydrolyzing and oxidizing influences from the environment, energy-rich bonds are maintained, so that an energetic gradient can be established. In addition, every organism can draw on a genetic and epigenetic system that originates from its evolution to establish an order to avert the tendency of its material components toward entropy. These principles enable the identity of the individual as well as that of the species. Information is the source for building up a higher degree of order than exists within the environment. Processing this information allows for *informational self-determination* and is part of autonomy.

Taken together, these are some features of autonomy: boundaries, metabolic processes, self-regulation, dynamic disequilibrium, energetic gradient, and informational self-determination, all of which need to be actively maintained.

An extensive study demonstrated that the ability for autonomy changed considerably during evolution (Rosslenbroich 2014, 2023b). The ability for regulation and stabilization of endogenous functions, as well as the flexibility within the environment, have been enhanced throughout evolution in certain lineages. In this sense, organisms were able not only to adapt to the environment, but also to expand their own individual autonomy.

Especially during the major transitions of evolution, the capacity for individual autonomy was gradually enhanced and extended. Enhanced autonomy is understood as a property that increasingly allows a system to maintain its functions in the face of internal and external perturbations and uncertainties, and at the same time extends the scope of self-generated, flexible reactions and active behaviors. Physiological stability and versatility lead to higher capacities of regulating life-functions and self-control of activities of the organism, which means greater resilience towards the environment. Generally formulated, it is a widening of possibilities. Thus, there are less-autonomous organisms that are more subject to the direct physical, chemical, and biological conditions of their surroundings, and more autonomous organisms that can act more on their own behalf because they are more active, flexible, and selective in their interaction with the environment.

These processes are changes in a relative autonomy, because numerous interconnections of the organism with the environment and dependencies upon it were retained simultaneously with the increasing autonomy.

To these changes in relative autonomy belong, for example, reinforcements of the external boundaries through the different types of epithelia and integuments, as well as fur and feathers, the concentration capacities of nephridial organs, homeostatic regulation capacities of circular systems, the evolution of high blood pressure systems, extension of movement capacities, and stabilization of body temperature, together with an extended aerobic capacity of the muscle system in endotherms and much more (for details see Rosslenbroich 2014).

The nervous systems in particular developed a basis to make possible more autonomous actions and reactions. The formation of more complex and concentrated brains has been extensively described in neurophysiology as encephalization (Striedter 2005). As a result, more and more flexible, self-determined behavior became possible in the course of evolution, and higher degrees of freedom of behavior emerged. Especially in humans, there was an extensive concentration of neurons in the brain and the development of areas of the cerebral cortex that are not monomodally fixed but available for multiple functions and creative combinations (Sherwood et al. 2012; Herculano-Houzel 2016; Kaas 2019). This flexibilization of behavior is an increase in the capacity of autonomy.

Thus, along the evolutionary series in autonomy, there appear additional forms of agentive behaviors which can be described as different levels of agency. In addition to simply maintaining the life functions, there are simple and then more complex behaviors of organisms. The crucial point is that organisms with extended capacities for autonomy may generate enhanced forms of agency.

## Levels of Agency

We propose to distinguish five levels of agency, but other classifications are also conceivable. This differentiation should allow some particular qualities of agency to be captured. However, the levels ultimately build on each other or are intertwined. It will be especially difficult to define clear boundaries between the last three levels (levels 3‒5), which are interactive activities in the environment. We therefore assume that there are fluent transitions between them. They are always connected to levels 1 and 2, which are constitutive processes of the organism and its ontogenetic changes. The proposed five levels are a first approximation to the existing phenomena.

*Organismic level of agency* (level 1) is the mere process of life and its functions involved. The maintenance of a metabolism, the production of proteins and of components of the membrane, as well as keeping the DNA intact and its transcription, are basic activities of an organism. This also includes the generation of seclusion from the environment, either by a membrane in single cells or by epithelial arrangements of tissues in multicellular organisms.

This level of agency is the basis for maintaining the integrity and processual functionality of the organism as such. It is a noncentric network of interdependent processes, integrated into a coherent, autonomous open whole. The agent is the whole organism (in the sense of integrative systems theory, see Noble 2006, 2017; Rosslenbroich 2023a). Potter and Mitchell (2022, p. 6) express it like this: “living organisms are dynamic, holistically integrated systems whose parts constantly act in concert, influencing and constraining one another in order to maintain the holistic pattern.”

The organismic level of agency is similar to the constitutive processes cited above (Arnellos and Moreno 2015; Moreno 2018). However, it differs from their definition in that we interpret these intrinsic processes of maintenance as agentic based on the examples mentioned above. These intrinsic processes have the capacity of self-maintaining and self-organization, which is unlike any machine. The processes show organismic self-activity. They are open and supportive for higher levels of agency, such as directed activity and locomotion. Further on, the intrinsic processes of maintenance are open and sensitive for behavioral changes of the individual, e.g., through epigenetic changes and plasticity (West-Eberhard 2003; Pigliucci and Müller 2010; Kümmell 2015).

Here we want to accentuate that this level of agency is conceivable as genuine without an external directedness, without a major goal, and of course without an intentionality directed into the future.

*Ontogenetic level of agency* (level 2): Like the former level, the ontogenetic level of agency is a noncentric network of interdependent processes, but contrasts to the former level in being directed. The goal of these processes is maturation with lifelong or with reduced growth. This level of agency predominates in the somatic development of multicellular organisms, but also appears in single-celled organisms, e.g., after cell division. In plants, it is expressed in a more or less successive appearance of organs (leafs, blossoms, and fruits). In animals, the organs develop approximately at the same time, as was already pointed out by the morphologist Goethe ([1790] 1981, p. 64).

Even when the organism is self-maintaining and self-organizing, it is of course not completely self-developing, because it is rooted through inheritance in its phylogentic past. However, as Walsh (2015, p. 181) pointed out: “The genome is no longer seen as embodying a program for building an organism. Rather, it is increasingly considered an ‘organ’, under the control of the cell and of the entire organism,” where epigenetic and intracellular genetic engineering processes occur (Shapiro 2013; Walsh 2015) and where living systems “interpret genetic information in a great diversity of context-dependent ways” (Sultan et al. 2022, p. 4). Thus, maturation is not a program, but an interdependent, active process of the organism itself, sensitive for the actual situation and the lifestyle of the individual.

*Directed agency* (level 3): This and the next levels of agency imply all activities of an individual connected to its environment including its own body (e.g., in grooming). This form of agency is centered insofar as the actions do not necessarily come from an interdependent network as in levels 1 and 2. They rather have a starting point in the individual, which can just act on its own causes. This, however, often takes place in the form of specific reactions to outer stimuli. The actions are directed, and on this level the goal of the action is necessarily connected to the action itself. This includes reflexes and instinctive, inherent behaviors without any decision of the animal.

When bacteria move in a certain direction, they measure the concentration, for example, of a nutrient substance, from one point to the next and follow a concentration gradient. To follow the gradient has a function: it is following a stimulus. However, even some form of decision-making has been described in bacteria (Adler and Tso 1974; Shapiro 2007; Schultz et al. 2009, 2013; Kalinin et al. 2010; Ben-Jacob et al. 2014; Mehdi Salek et al. 2019; Park and Aminzare 2020). Schultz et al. (2009, p. 21,028) wrote: “We see that the bacteria’s decision relies on a combination of deterministic and stochastic events processed by many modules that sense information, process information, and regulate cell responses.” And Potter and Mitchell (2022, p. 7) mention: “contextual information about the current metabolic state of the cell constantly integrates with and modulates the chemotactic pathway… in a way that is highly nonlinear.” Although decision-making is present in a primordial way, it differs greatly from the form of decision-making of animals in which higher grades of behavioral flexibility evolved, which is described with the next level.

*Directed agency with extended flexibility* (level 4): This form of agency is centered as is the former, but this level represents more complex forms of behavior, in which the individual has the ability to choose from different options. The action and the goal are not directly connected as in level 3. Rather a higher flexibility evolved to reach the goal.

The animal has the choice of different pathways to reach the goal and needs to make decisions. The goal can be modified during the process of action, insofar as the action can be interrupted, e.g., when there are too many obstacles or an alternative choice can be followed when the first choice did not succeed. In the terms of Tomasello (2022) this form of agency can be called psychological agency, because it is under the individual’s flexible control.

When looking at the flexibility in the hunting performance of a lion, level 4 is obviously part of it, although level 3 may also play a role, e.g., when hunger triggers the lion to start hunting. The lion follows his goal through many actions, combining many behaviors and complex activities in order to achieve its aim. Its movements need a certain form of mental representation, which guides the different actions. Thereby the elements of the behavior and the forms of movement can be combined flexibly. In the decision process, the individual’s ability to control its actions (Tomasello 2022) and its experiences play a role.

Delafield-Butt (2021, p. 82) stresses the role of feelings in decision-making: “We evaluate affordances for action and interaction in the world and generate feelings about the best course of action. These evaluative feelings are brainstem-mediated, part of the most phylogenetically ancient neural system that governs all animal action.”

Especially the psychological level of agency is of a rhythmic nature. The other forms of agency usually show rhythms according to the respective processes too (see above), but in the psychological level of agency the rhythm is very obvious, because animals show phases of inactivity. They sleep, for instance, or just rest. However, that does not mean that agency is absent in the inactive phases. It is at least present as a potential. A sleeping animal can suddenly wake up even in its normal sleeping phases and all its abilities of flexible (re)actions reappear. Resting, on the other hand, can also be termed “active passiveness.” Barandiaran et al. (2009) wrote that kittens brought closer by their mother and warmed up by her are not agents in that action, because it is the mother who is driving the coupling. However, the passiveness of the kittens is also part of their agency, because they like to be close to their mother and feel her warmth, so they want to stay (the goal is to stay). They have a choice; they have the potential to run away (when they are not too small), but they stay. The decision not to act in a certain situation is also a sort of psychological agency. Or even in “passive actions” like falling off the roof, the organism is still acting, because there are emotional reactions and very likely the effort to fall as favorably as possible. Cats have the ability to turn around when falling, so that they usually end up on their legs, even when they were upside down when the fall started (Bischoff 2023). So even in passive situations or within unforeseen accidents, the animal still behaves as an agent.

Ontogenetically, level 4 agency may show a long phase of development, in which the animal learns to perform certain actions.

*Agency with a preconceived goal* (level 5): This form clearly requires a different level of abilities and is only possible within a complex organism with a highly developed central nervous system. Humans especially, and in initial stages also some higher animals, can act according to an envisioned goal, which can be kept in memory and processed over a long time.

This needs to be distinguished from the situation of the lion. The goal, to catch prey, must be present for the lion—it must be part of its current situation. This includes being hungry, for example. However, it will not plan to hunt the day after tomorrow when it will be hungry again, while he is satiated today. Such a form of planning, which can be decoupled from the current situation, is regularly involved in human actions.

The case of squirrels burying nuts in order to keep them for the winter is different from the situation in humans, as it is assumed that this behavior follows a fixed action pattern rather than a preconceived goal (Eibl-Eibesfeldt 1999).

Humans regularly plan for the future and arrange things long before they become a reality. We can mentally hold goals for shorter or even very long times, and we can develop many techniques to follow this goal. The basis is a mental representation of a plan, which can be brought about quite independently of the current situation, and which is accommodated and creatively changed by the ability of thinking and creative fantasy. Humans are able to decide by considering mentally represented alternatives. This ability is likely to have developed only slowly in the course of human evolution.

In behavioral research, it is difficult to tell in many cases whether this form of planning is already initially available to some animals. For example, chimpanzees have regularly been observed preparing a stick that is used to peck for termites much later and in a completely different location. From experiments with the more intelligent primates and some birds, such as different species of ravens and parrots, there is much evidence for such abilities. Nevertheless, clearly the planning behavior of humans far exceeds any of these forms of behavior.

Agency with a preconceived goal further evolved in the genus *Homo*, when different members of one group started to work together with a joint goal in a shared world. This form of social agency is based on the ability to communicate with another person at least to some degree, to have some sort of social understanding of the actions of other persons and to act in joint attention in the group (Laland 2017; Tomasello 2022).

Agency level 5 needs a long phase of development, especially in humans, in which ontogenetic stages are prolonged during the whole hominine evolution (Gould 1977; McKinney 2002). Flexible learning especially provides the prerequisites for planning abilities.

## Stages in the Evolution of Agency

There have been several major transitions which opened up new evolutionary pathways after their implementation (Kümmell 2011, 2020; Rosslenbroich 2014). These include the evolution of multicellularity, the conquest of land, the gain of endothermy, becoming parasagittal in stance and gait, becoming upright with bipedal stance and gait, and the evolution of the human brain, all having some impact on the evolution of the different levels of agency.

Unicellular organisms predominantly show level 1 (organismic agency) and level 3 (directed agency). They maintain themselves through their cell processes and interactions with the environment, which predominantly are reactions to stimuli. However, as mentioned before, decision-making processes of bacteria have been recorded, so that their behavior cannot be completely reduced to simple reactions to the environment. The directed processes of ontogenetic growth (agency level 2) appear in unicellular organisms after cell division, but also in a more complex manner by budding or sexual reproduction. For instance, the unicellular Suctoria, which are sessile Ciliata, are known to reproduce by budding, where the buds are released as swarmers. After settlement, they grow and develop to a final stage with tentacles and a stalk. In, for example, Foraminifera and Ciliata, sexual reproduction is possible. Two haploid gametes copulate and form a zygote, followed by growth (Westheide and Rieger 1996). Thus, ontogenetic agency is already involved in some groups of unicellular organisms and therefore may have been present in the Precambrian.

According to the molecular clock, animal multicellularity evolved by ∼ 800 million years ago, and in the “Cambrian explosion” at ∼ 541 million years ago nearly all stem phyla of animals appeared in the fossil record, including real vertebrates (Shu et al. 1999, 2003). This has been described as different combinations of organismal resources of autonomy (Rosslenbroich 2014). With the evolution of somatic cells, agency level 2 was further developed. Developmental processes with differentiated cells evolved as well as several ontogenetic stages such as a larval stage, a stage of sexual maturity, and an adult stage, often accompanied by considerable morphological changes. Agency level 1 (organismic agency) also changed during the transition to multicellularity, extending its scope from cell biology to physiology. While biochemical and genetic core processes have been conserved, novelties evolved in the realm of cell differentiation, developmental processes, and the different body plans (Kirschner and Gerhart 2005).

Within a tissue of a multicellular organism, single-cell behavioral agency (level 3) is largely lost or transformed into collective modes of behavior within the context of the tissue and the whole organism (Newman 2023). Interestingly, Newman (2023) reported experiments that show that cells taken from normal tissue can navigate as single cells in mazes and make decisions, which are productive for their survival. Thus, the cells even in tissues keep a latent potential for behavioral agentic activities. Within the tissue, however, the single cells behave in the context of the whole organism, being constrained by the system. Sonnenschein and Soto (1999) proposed an interesting theory, which assumes that proliferation and activity is an in-built property of all cells and that a multicellular organism needs to control it. Even when the individual agentic behavioral abilities of the cells are reduced within multicellular organisms, the agentic capacities of the whole organism can be extended.

The transitions of the evolution of endothermy show how flexibility increases within agency level 4 (directed agency with extended flexibility) in the course of a very long timescale. The evolution of whole-body endothermy is known from land vertebrates, in sauropsids on the line to birds, and in synapsids on the line to mammals. The ocean does not facilitate the evolution of whole-body endothermy. That is very likely because the temperature differences are not as high as on land and the gills of fish do not transport enough oxygen for an endothermic organism. However, regional endothermy, where certain organs show thermoregulation, does occur (Grigg et al. 2022). The timescale in which endothermy evolved is still under discussion. Grigg et al. (2022) suggest that the evolution of endothermy pre-dated the divergence of sauropsids and synapsids in the Carboniferous. This analysis is based on fossil bone histology. Besides bone histology, there are also many direct and indirect (reconstructed) indications in the skeleton suggestive of endothermy, although most of them are not unequivocal. Such indications are the presence of a secondary palate, a diaphragm, maxilloturbinalia, the loss of the pineal foramen, brain expansion, increasing parasagittality, and a high metabolic rate. In synapsids, these features evolved in a complex mosaic pattern within the timeframe from the Permian up to the Middle Jurassic (Newham et al. 2020; Laaß and Kaestner 2023). Thus, the evolution of endothermy very likely appeared in a long-lasting trend with different grades of endothermy, during which the animals became more flexible and active by achieving thermal independence from the environment.

Endothermy, parasagittal movement, a relatively big brain with an expanded cortex, and many other properties led over long timescales to a mammalian organization with a predominantly very active, flexible, and highly sensitive life style. On the basis of this organization, humans considerably expanded the scope of self-determined activity through the dimension of thinking in terms of planning, temporal integration, and collective activity in groups and societies, so that a stage became possible that integrates the more basic levels, but also extends to a fully developed level 5. Thus, overall, the evolution of different stages in activity correlates with autonomy capacities.

## The Value of Differentiation

Level 5 is the one that is predominantly used in general philosophical discussions of agency (Schlosser 2015). It includes a background of desires, beliefs, intentions, and especially of mentally preconceived goals, as we are used to having them in human actions.

However, as we have shown, there is a broad spectrum of organismic activities without preconceived goals or even flexible behaviors and intentions. In level 4, some forms of purpose or intention are involved. When a bird flies to the bird house, it has the intention to feed there, and it knows from memory where it is. A marmot whistles with the intention to warn buddies about approaching danger. But there is a continuum between noncentric, basic levels of activities that serve merely to sustain life and more complex forms of activity. In many cases it is difficult to decide which level may be involved, and a classification also has its limitations.

The distinction between different levels of agency may help to avoid inappropriate connotations describing agency. It is possible to describe agency without anticipated final states of the activity. Animals, for example, even more complex ones, mainly live in the present. If they are hungry, they look for food, but they don’t imagine the necessity of caring about food tomorrow. For plants this seems to be even more true. To free the phenomenon of agency from an anticipated goal in any case makes this essential property of living organisms negotiable for science. The human version of agency needs not to be introduced into the behavior of other organisms (Hoffmeyer 2013).

This also touches the debate about teleology, a debate that has been conducted quite extensively throughout the history of biology (Brandon 1981; Di Paolo 2005; Corning et al. 2023). It is burdened too much by the assumption that the description of a behavior or a function as being teleological would in any case include the expectation that a goal in the sense of level 5 is expected. Also, when organisms are described as being active during evolutionary changes, this does not include a goal for the overall process.

If one differentiates, descriptions are possible without assuming such goals in each case. Many functions and behaviors of organisms are based on agency without an imagined future state. In many cases it “just happens”—it is just the activity within the present situation. As humans, we are used to our ability to envision a future goal, but this is not the case in most other forms of nature.

Biology never got rid of teleological descriptions and formulations, although at times the field tried hard to rely solely on explanations of cause and effect, in which there is no room for any agential explanation. The proposed differentiation may help to adjust the understanding of underlying phenomena.

### Psychological Agency

Tomasello (2022) recently published *The Evolution of Agency*, in which he also developed different levels of agency. However, his focus is especially on psychological agency, while he explicitly excludes an organismic view of the phenomenon. For him, simple forms of agency begin with reptiles, which are then complemented to mammals, including humans, in further forms.

Basically, we agree with the formulation of different levels of agentive behaviors. Nonetheless, we would like to draw attention to the fact that a psychological agency always presupposes a living organism that makes this self-activity possible. The biological level must therefore also be taken into account. Additionally, we don’t see a reason to start with reptiles. There are many fish and even more primordial animals with relatively sophisticated behaviors, so that it is hard to draw a line. Also, within invertebrates there are examples of sophisticated behavioral possibilities. But there is an overlap of Tomasello’s “goal-directed agents” and “intentional agents” with our level 4. And his “rational agents” and “normative agents” of humans overlaps with our levels 4 and 5.

As a spectrum of different abilities can be observed, it is difficult to make strict demarcations here. Nevertheless, it is important not to lump everything together, and no doubt there are different ways to classify and describe the different levels and abilities. However, we see it as essential to locate these capacities much more fundamentally in biological processes, and we see their origin at the origin of life itself.

Another point is that what Tomasello describes as levels of agency is similar to our proposed stages of autonomy capacities. If autonomy theory is regarded, the described abilities can be located much more comprehensively in biological organization.

### Agency in Evolution

Walsh (2015) argues that agency is also a central element to actuate the evolutionary process. He explains that the category “organism” has played only a small role in evolutionary theory so far. Conventional theory principally deals with the dynamics of supraorganismal assemblages (populations) of suborganismal entities (genes). The primary agency of the whole organism, as a distinct property that is able to make differences, plays virtually no part in the explanation of evolutionary phenomena within the synthetic theory of evolution that has grown to such prominence throughout the 20th century. This has not gone unnoticed, Walsh (2015) writes, and cites Goodwin (1994, p. 1): “Something very interesting has happened to biology in recent years. Organisms have disappeared as the fundamental unit of life. In their place we now have genes, which have taken over all the basic properties that used to characterize living organisms.” And then, of course, he cites Dawkins (1976, p. 82), who took this view to the extreme:

Evolution is the external and visible manifestation of the survival of alternative replicators.… Genes are replicators; organisms… are best not regarded as replicators; they are vehicles in which replicators travel about. Replicator selection is the process by which some replicators survive at the expense of others.

Walsh argues for an organism-centered alternative to this view, since organisms participate in evolution as agents. Similar views have now been formulated by many authors (see, e.g., Turner 2007; Shapiro 2011; Kümmell 2015; Aaby and Desmond 2021; Sultan et al. 2022; Ball 2023a, b; Corning et al. 2023; Noble and Noble 2023). Kirschner and Gerhart (2005, p. 252), for example, formulated: “On the side of generating phenotypic variation, we believe the organism indeed participates in its own evolution.”

## Conclusion

Our proposal includes three main points:

1. Agency is a general principle of being alive. Even the basic life processes such as metabolism, information processing, and the formation of organic matter are an intrinsic activity and can in this respect be described as agency. We called this basic principle “organismic level of agency.” Being alive is identical with being (periodically) active.
2. Several levels of agency can be described. They capture different qualities that occur or are transformed during evolution. In addition to the organismic level, we propose an ontogenetic level, directed agency, directed agency with extended flexibility, and a level that includes the capacities to follow preconceived goals. It need not necessarily be this division into five levels; other differentiations are conceivable. However, a general distinction and a description of the different qualities, which are exhibited in the organic world, are crucial to gain a more precise understanding of what is indicated by agency.
3. The two organismic properties, autonomy and agency, are closely related. Enhanced physiological and behavioral autonomy extends the scope of self-generated, flexible reactions and actions. The increase in autonomy through the evolution of a widened scope of behavioral possibilities and versatility in organisms coincides with extended levels of possible agency. An organism that exhibits agency of level 4 or 5 needs a complex, highly developed physiological organization and especially a complex nervous system with a sophisticated brain.

Human organization especially, including the sophisticated brain, is the basis for extended levels of agency with the capacities to follow preconceived goals. However, it is important for the understanding of the basic principle of agency not only to refer to this form in behavioral and organismic explanation, but to differentiate.
